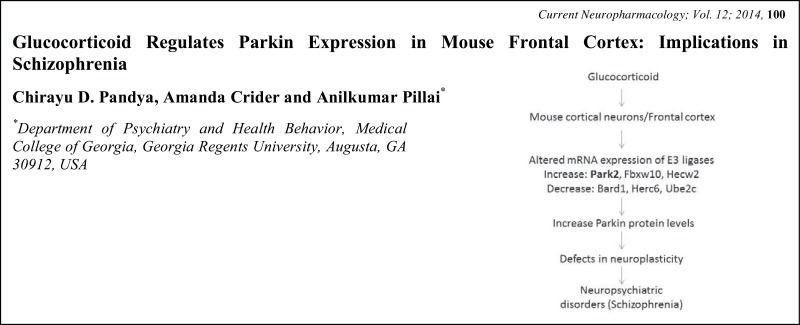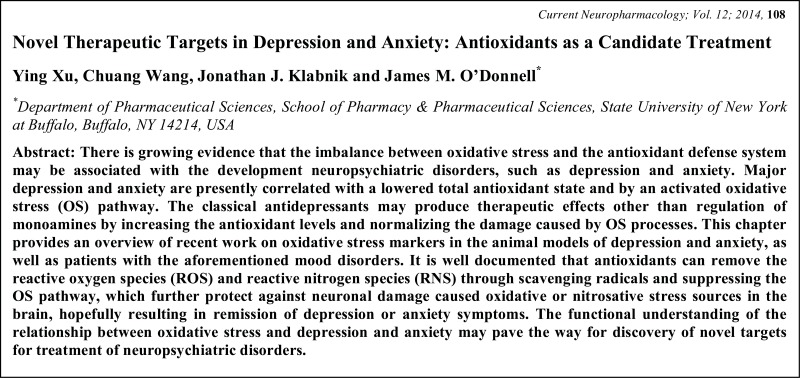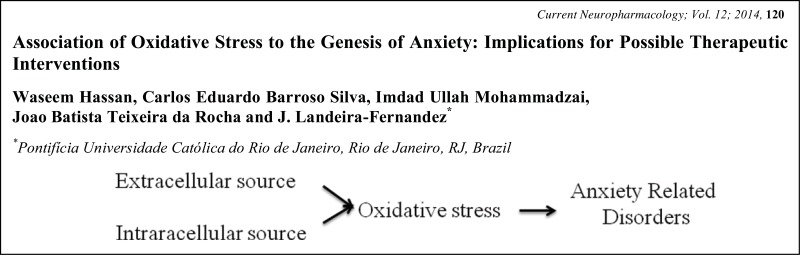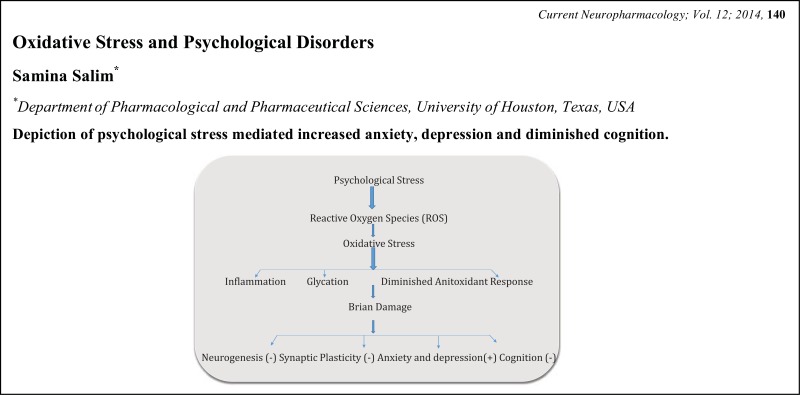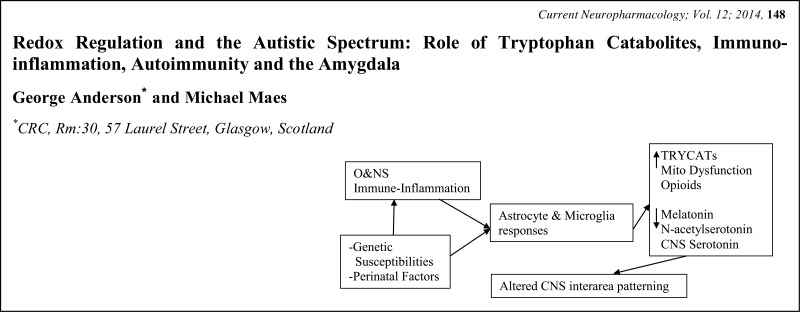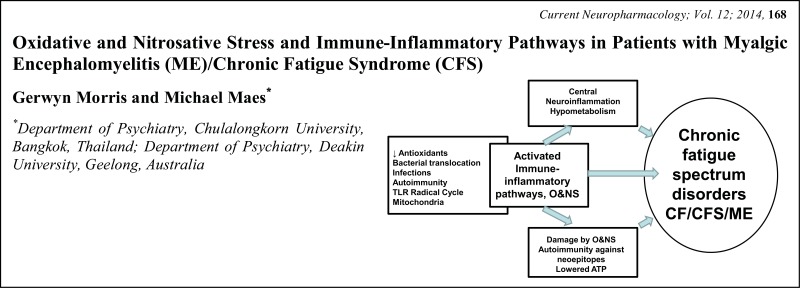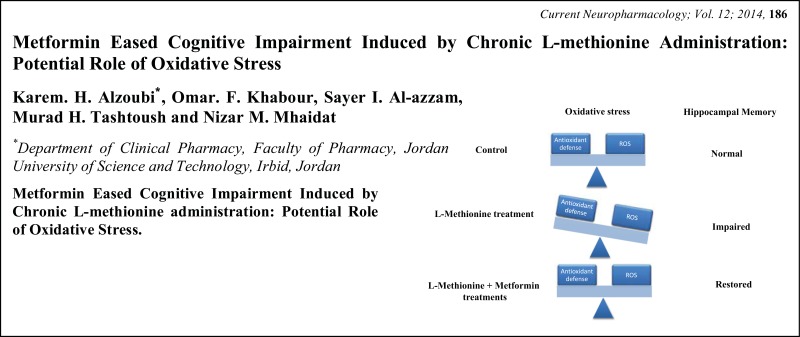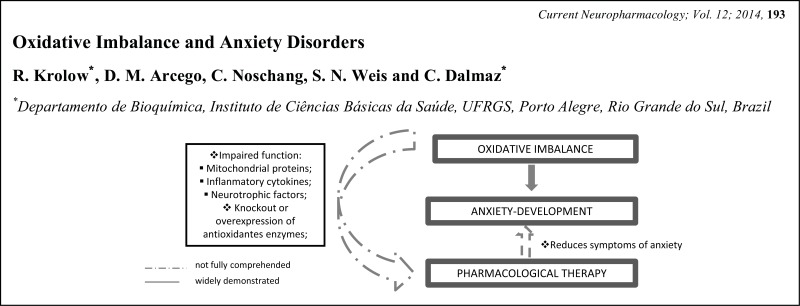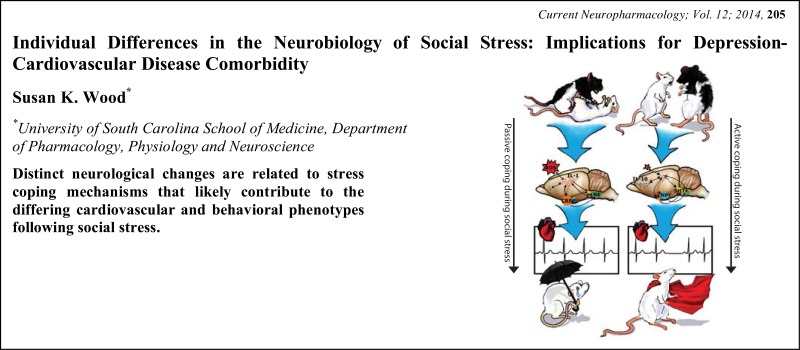# Graphical Abstracts

**DOI:** 10.2174/1570159X1202140307121408

**Published:** 2014-03